# A pilot study on pupillary and cardiovascular changes induced by stereoscopic video movies

**DOI:** 10.1186/1743-0003-4-37

**Published:** 2007-10-04

**Authors:** Hiroshi Oyamada, Atsuhiko Iijima, Akira Tanaka, Kazuhiko Ukai, Haruo Toda, Norihiro Sugita, Makoto Yoshizawa, Takehiko Bando

**Affiliations:** 1 Division of Integrated Physiology, Niigata University Graduate School for Medical and Dental Sciences, Asahi-machi1, Niigata, 951-8510, Japan; 2 Department of Human Support System, Faculty of Symbiotic Systems Science, Fukushima University, Fukushima, Japan; 3 Department of Applied Physics, Waseda University, Tokyo, Japan; 4 Research Division on Advanced Information Technology, Information Synergy Center, Tohoku University, Sendai, Japan

## Abstract

**Background:**

Taking advantage of developed image technology, it is expected that image presentation would be utilized to promote health in the field of medical care and public health. To accumulate knowledge on biomedical effects induced by image presentation, an essential prerequisite for these purposes, studies on autonomic responses in more than one physiological system would be necessary. In this study, changes in parameters of the pupillary light reflex and cardiovascular reflex evoked by motion pictures were examined, which would be utilized to evaluate the effects of images, and to avoid side effects.

**Methods:**

Three stereoscopic video movies with different properties were field-sequentially rear-projected through two LCD projectors on an 80-inch screen. Seven healthy young subjects watched movies in a dark room. Pupillary parameters were measured before and after presentation of movies by an infrared pupillometer. ECG and radial blood pressure were continuously monitored. The maximum cross-correlation coefficient between heart rate and blood pressure, ρ_max_, was used as an index to evaluate changes in the cardiovascular reflex.

**Results:**

Parameters of pupillary and cardiovascular reflexes changed differently after subjects watched three different video movies. Amplitudes of the pupillary light reflex, CR, increased when subjects watched two CG movies (movies A and D), while they did not change after watching a movie with the real scenery (movie R). The ρ_max _was significantly larger after presentation of the movie D. Scores of the questionnaire for subjective evaluation of physical condition increased after presentation of all movies, but their relationship with changes in CR and ρ_max _was different in three movies. Possible causes of these biomedical differences are discussed.

**Conclusion:**

The autonomic responses were effective to monitor biomedical effects induced by image presentation. Further accumulation of data on multiple autonomic functions would contribute to develop the tools which evaluate the effects of image presentation to select applicable procedures and to avoid side effects in the medical care and rehabilitation.

## Introduction

Taking advantage of recent developments in the image technology, new trials of efforts to promote health by utilizing images are expected. Images may be applied to the medical care, or may be used as the tools to monitor the effects of the care. One of the prerequisite of these trials is the understanding of biomedical influences evoked by visual stimulation. The biomedical influences evoked by presentation of images have been studied in efforts to prevent biomedical hazards such as asthenopia and other symptoms of the VDT syndrome evoked by using video displays in the business offices [1-6]. Results of these studies indicated that autonomic responses, including cardiovascular and ocular responses, would provide valuable information.

In this study, changes in the pupillary light reflex and cardiovascular reflex evoked by watching three different stereoscopic video movies were measured in healthy young subjects, and related with the subjective assessment of discomfort measured as scores of the questionnaire collected at the same time. It is shown that biomedical effects evoked by presentation of video movies were different depending on the properties of video movies. Possible causes of these differences are discussed. Accumulation of the knowledge may provide the efficient tool to select proper images applicable to the cases, and to evaluate properly the effects of treatments in the field of medical care and rehabilitation. Such estimation is also necessary to avoid the side effect or aggravation due to improper stimuli. Some of the preliminary data were reported in the abstract form [7].

## Methods

### Subjects

Subjects were seven (five male and two female) medical students (23.0 ± 0.9 years). The procedures and general purpose of the experiments were explained to subjects, but no information on the expected results was given. The Bioethics Committee of the Niigata University School of Medicine approved the experiments in this study, and all subjects gave the informed consents to participate in the study.

### Presentation of motion pictures

Three stereoscopic movies of 5-min-long were used as the test stimuli. The digital signals of the movies were fed to a liquid-crystalline display, and total brightness of a frame in the movie was monitored by a photocell on the screen, which was positioned in front of the display. The binocular disparity was roughly evaluated by the MATLAB software (MathWorks, Inc), in which the separation of the central objects in even and odd frames of the movie was calculated.

Among three movies, two were made of computer graphics (CG), and the other was the real scenery taken by a camera in a car of the roller coaster (R), which gave strong vection sensation in all subjects, probably because the quick changes in the apparent velocities of objects in the scenery would invoke the past experiences of subjects. One of the CG movies was an imaginary work, in which various objects were moving violently without a consistent story through the movie (movie A). The other CG movie dealt with an imaginary ancient world in which many kinds of dinosaurs approached the subjects with the progression of the story, and finally the subject was attacked by a tyrannosaurus (movie D).

Other properties of the movies were as follows. Firstly, brightness in two CG (A and D) movies was changed frequently. Their mean brightness was in the same range, but switching in the brightness was much frequent in the movie D. The movie R had stable and high brightness. Secondly, the degree of binocular disparity is larger in two CG movies (largest in the movie D) than in the movie R. Thirdly movies D and R had a kind of story, which proceeded from the beginning to the end, while blocks of frames were not temporally continuous in the movie A.

Subjects watched three different video images in a random order in a day. Before the presentation of each video movie, five minutes of rest were allowed for each subject, which were necessary to prepare the stable condition of subjects, and to collect stable cardiovascular data as the control. Just after presentation of the movie, measurement of pupillary parameters was quickly performed. Then, five minutes of the rest were again allowed to collect cardiovascular data. The same subjects repeated the experiments within two weeks at the corresponding time zone in each day to avoid the influence of the circadian rhythm of pupillary parameters [8].

**Figure 1:**
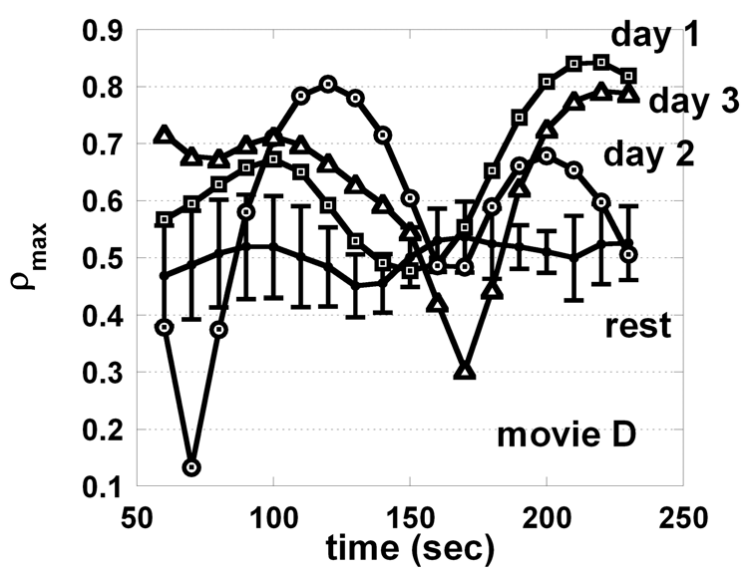
The ρ_max _of a subject obtained in the consecutive three days (day 1, day 2 and day 3) during video presentation of the movie D is shown respectively. The mean and SE of ρ_max _obtained in the rest before and after presentation of the movies ρ_max_control _are also shown (control, n = 6).

Stereoscopic movies in digital video cassettes were consisted with the sequential frames of odd and even fields, which provided images to the left and right eyes with the binocular disparity necessary for stereoscopic vision. They were replayed by a video cassette recorder (WV-D10000, Sony Co.), fed to a signal distributor, and then were rear-projected by two aligned liquid-crystalline display (LCD) projectors (TH-L795J, Matsushita Elec. Co., XGA, total of 1400 lm) onto an 80-inch screen (Fig. 1). By an electronic distributor, even and odd fields of the images were allotted to each of the two LCD projectors. Each of two projectors had a polarizing filter, orthogonal to each other. Subjects sat in a chair at 2 m from the screen, wearing polarizing-glasses and watching motion pictures in the 80-inch screen, with the comfortable posture in the dark room (illuminance, 10 l× at the floor of the room just in front of the screen). The size of the images in the screen was 120 cm (length) × 160 cm (width). The visual angle was vertically 17 deg, and horizontally 22 deg, because the distance between the subject and the screen was 2 m.

### Measurement of pupil diameter

Pupil diameters were measured by an infrared pupillometer (Iriscorder C7364, Hamamatsu Photonics Co.), in which a charge-coupled device (CCD) camera took the image of a pupil with a sampling rate of 1/60 sec. This camera had an effective field of 30 mm × 22.5 mm. The field was illuminated by a light-emitting diode (LED) with a peak wavelength of 890 nm. Another LED in the pupillometer (peak wavelength, 660 nm; maximum intensity, 10 μW) was lit for 1 sec to induce the pupillary light reflex. Measurements of the pupil were performed in the dark room (illuminance, 10 lx). In the control, the parameters of the pupil were measured after the rest of 5 min in the dark room. After presentation of movies, they were measured just after presentation in the dark room. Then the difference in the brightness of video movies might contribute to the differences in the pupillary parameters, but it was not the case in this study (see Discussion, changes in pupillary parameters). Data were collected by an interface (PCI-MIO-16XE-10, National Instruments Co.) by the aid of the LabVIEW (National Instruments Co.) and stored in a hard disk. Original data were also stored in a digital tape by using a data-recorder (RD135T, TEAC Co.).

A polynomial curve was fitted to the rising or falling time course of the pupillary light responses. The maximal velocity and acceleration of pupillary constriction, and the maximal velocity of re-dilation of the pupil in the light reflex were calculated by the first- and second-order differentiation of the fitted curve.

Amplitudes of the pupillary light reflex are dependent on the pupil diameter before light stimulation (baseline pupil diameter, D1). We then adopted the constriction ratio [9], CR, to balance the differences in D1 as follows: CR = (D1–D2)/D1, where D2 was the diameter of the pupil at the peak of the light reflex. The CR_ratio _was defined as (CRaf/CRbf), to evaluate the changes in CR before (CRbf) and after (CRaf) presentation of movies.

### Measurement of blood pressure and ECG

ECG (electrocardiogram) (Nihon-Koden Co.) and radial blood pressure (JENTOW770, Colin Japan Co.) were continuously collected by a data-collection system at a sampling rate of 1 kHz with 12 bit resolution. Data in the rest time before and after video presentation (5 min each), and those during video presentation (5 min) were analyzed. Heart rate (min^-1^) was calculated from the reciprocal of the inter-R-wave interval of the ECG signal. Mean blood pressure (mmHg) was obtained as the mean value of the pressure signal over one heartbeat. Beat-to-beat mean pressure and heart rate were interpolated by a cubic spline function and were re-sampled every 0.469 sec to yield corresponding beat-to-beat data, denoted by *BP *and *HR*, respectively. The data is filtered through a band-pass filter with the bandwidth between 0.08 Hz and 0.12 Hz to extract Mayer wave components. At time *t*, Hanning window whose interval is [*t*-60, *t*+60] in second is used to segment *BP *and *HR *into 2 min-long data. After this processing, the normalized cross-correlation function ρ(τ) between *BP *and *HR *is calculated. The ρ_max _was defined as the maximum cross-correlation coefficient ρ(τ) for the positive τ [10,11]. The ρ_max _would be 1, if changes in the heart rate depend completely on changes in blood pressure. But it is ordinarily lower than 1, because the heart rate depends also on the biological noises embedded in the baroreflex loop. When noises are increased, for example, by emotional inputs, ρ_max _is lowered. The ρ_max _would be also lowered, if the vascular resistance changes without the corresponding change in pulse rate. On the other hand, increased ρ_max _would be induced by the reduction of biological noises. The contribution of noises may be lowered by the stimuli that drive cardiac reactions to prepare movements of the body.

### Data analysis

Subjects evaluated their physical conditions during the rest before and after presentation of movies by filling out a questionnaire with 10 items on a seven-point rating scale [12].

We used the paired *t*-test (two tailed) to compare means. Pearson's correlation coefficient was used to assess the relationship between two parameters. We used the SPSS software (release 10.07J, SPSS, Inc.) for statistical analyses.

## Results

The scores of the questionnaire (the last column in Table 1) increased significantly after the subjects watched any of three video movies (*p *< 0.02, for movie D, and *p *< 0.05, for movie A and R, paired *t*-test), indicating that they felt some discomforts by watching 3D movies or possibly by restriction of body movement with various equipments in the experiment.

**Table 1:** The pupil parameters and the scores of questionnaire obtained before (control) and after presentation of movies A, D and R

	**D1 [mm]**	**D2 [mm]**	**CR**	**latency [msec]**	**vc [mm/sec]**	**vd [mm/sec]**	**ac [mm/sec2]**	**peak time [sec]**	**score of questionnaire**
**Control**	6.62 ± 0.89	5.25 ± 1.03	0.21 ± 0.07	303 ± 29	3.99 ± 1.29	1.14 ± 0.36	32.1 ± 11.3	1.10 ± 0.24	32.4 ± 10.2
**movie A**	5.95 ± 0.98**	4.48 ± 1.06**	0.25 ± 0.09*	304 ± 28	4.33 ± 1.27	1.14 ± 0.42	34.8 ± 12.7	1.10 ± 0.27	36.1 ± 10.3*
**movie D**	6.08 ± 0.92**	4.54 ± 0.93**	0.26 ± 0.08**	300 ± 32	4.35 ± 1.11	1.16 ± 0.48	32.3 ± 9.6	1.10 ± 0.20	38.0 ± 14.1**
**movie R**	6.15 ± 0.86**	4.77 ± 0.92**	0.23 ± 00.8	306 ± 33	4.02 ± 1.37	1.10 ± 0.43	32.2 ± 11.5	1.06 ± 0.18	36.7 ± 11.3*

mean ± SD. D1: baseline pupil diameter just before light stimulation to induce the light reflex (mm), D2: pupil diameter at the peak of the light reflex (mm), CR: the amplitude of the pupillary light reflex (D1–D2) divided by D1, latency: the latency of the pupillary light reflex (msec), vc: the velocity of constriction (mm/sec), vd: the velocity of re-dilation (mm/sec), ac: the acceleration of the constriction (mm/sec^2^), peak time: time at the peak of the pupillary light reflex (sec), and the scores of the questionnaire. *, *p *< 0.05, **, *p *< 0.01. Paired *t*-test (two-tailed).

### Pupillary parameters

Seven subjects watched three different video movies in a random order in a day, and the test was repeated two times within two weeks. Then total of 21 trials for each of three movies was performed. The changes in data obtained in the day1, day2 and day3 were not different each other (ANOVA, LSD and Bonferroni), and therefore, data in these 21 trials were pooled. The baseline pupil diameters (D1) were measured just before the onset of light stimulus which induced the pupillary light reflex. The D1_video, which was obtained after presentation of video movies, was significantly smaller than the D1_control, which obtained before presentation in all of three movies (Table 1). In addition, values of the D1_video for any of three video movies were not significantly different each other. The pupil diameters at the peak of the light reflex (D2) were also significantly smaller after presentation of movies.

The constriction ratio of the light reflex, CR, was significantly larger after presentation of the movies D (*p *< 0.01, paired *t*-test) and A (*p *< 0.05) than the control obtained before presentation (the control) (Table 1), while after presentation of the movie R the CR was not significantly different from the control (*p *> 0.05). Other parameters of the pupillary light reflex, i.e., the latency of the constriction, the velocity of constriction (vc), the velocity of re-dilation (vd), the acceleration of constriction (ac), and the time at the peak of constriction (peak time) were not significantly different before and after presentation of movies A, D and R.

### Cardiovascular reflex

Heart rate and blood pressure were continuously monitored. The ρ_max _was calculated for 2 min. Then the data window was shifted by 10 seconds, and the ρ_max _was again calculated. In this way, 18 points of the ρ_max _were obtained between 60 and 230 seconds following the onset of the movie. In Fig. 1, the values of the ρ_max _measured when a subject watched movie D in the first, second and third days are plotted against the time after the onset of the movie.

To evaluate the changes in the ρ_max_, the ρ_max_ratio _was defined. Firstly the ρ_max _at each of the 18 points along the time following the onset of a movie was calculated (ρ_max_test_). Secondly the values of ρ_max _at the corresponding points in the rest before and after presentation were averaged (ρ_max___control_). The standardized ρ_max _is defined as the ρ_max_test_/ρ_max_control _at each of 18 points. Thirdly the mean of the standardized ρ_max _for 18 points gave the ρ_max_ratio_. In Table 2, the mean ρ_max_control_, the mean ρ_max_test _as well as the ρ_max_ratio _are shown. The mean ρ_max_test _was significantly larger than the mean ρ_max_control _when the subject watched the movie D (*p *< 0.05, paired *t*-test), while the mean values of the ρ_max_test _were not significantly different after presentation of movies A and R (*p *> 0.05).

**Table 2:** Mean values of ρ_max_control_, ρ_max_test_, and ρ_max_ratio _are shown for each video movie (movie A, D and R)

	**ρ_max_control_**	**ρ_max_test_**	**ρ_max_ratio_**
**Movie A**	0.65 ± 0.10	0.66 ± 0.13	1.03± 0.06
**Movie D**	0.66 ± 0.12	0.70 ± 0.08*	1.11± 0.05
**Movie R**	0.66 ± 0.09	0.69 ± 0.12	1.08± 0.07

mean ± SD. *, *p *< 0.05. Paired *t*-test.

### Correlation between objective and subjective indices

**Figure 2:**
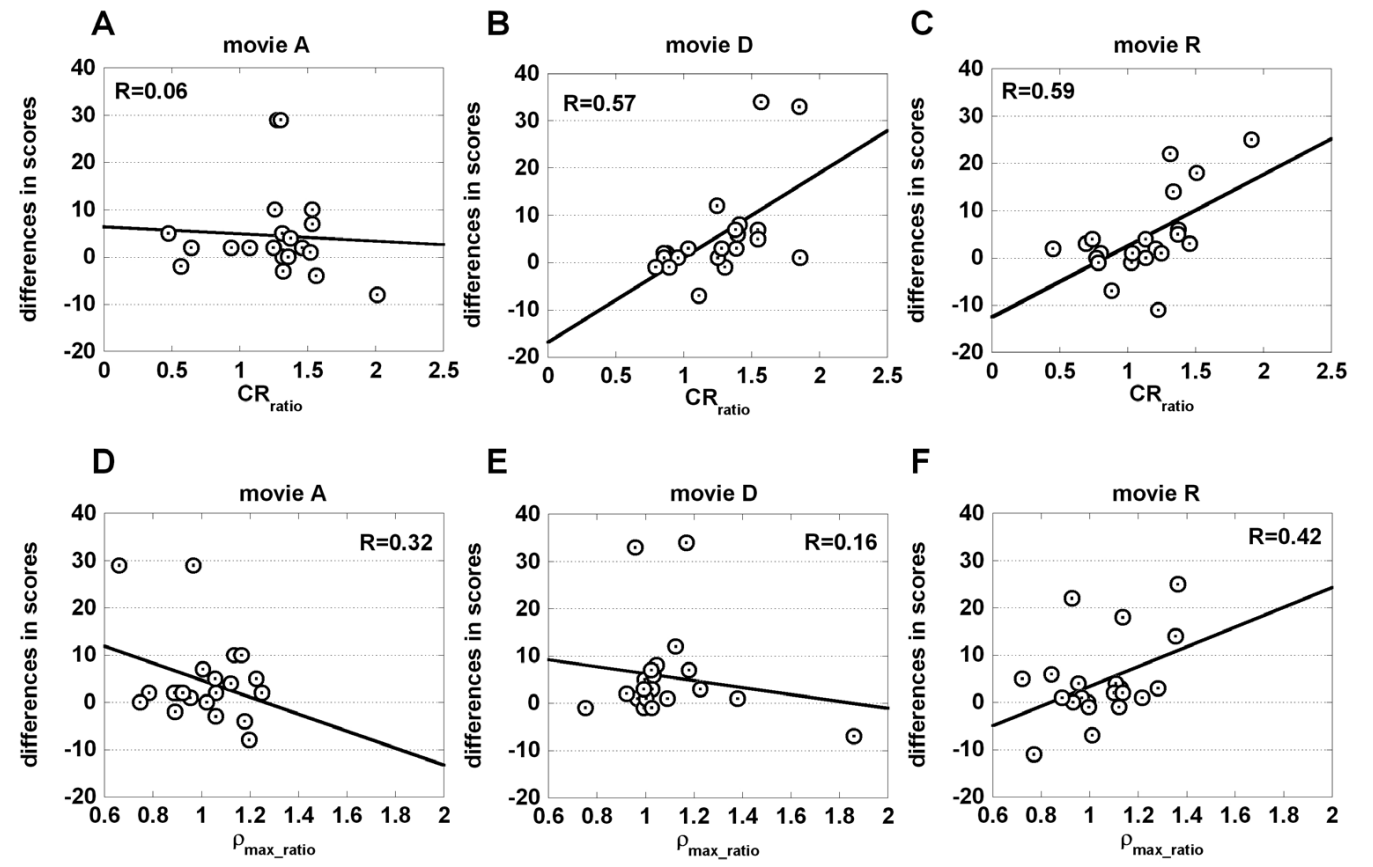
Correlation of pupil and cardiovascular parameters with the scores of questionnaire. A-C. Correlation of CR_ratio _with the differences in the scores of questionnaire when subjects watched the movies A, D and R, respectively, are shown. D-F. Correlation of ρ_max_ratio _with the differences in the scores of questionnaire when subjects watched the movies A, D and R, respectively, are shown.

The CR_ratio _obtained after the subject watched movie D or R was correlated significantly with the difference in the scores of questionnaire (*p *< 0.01) (Table 3, Pearson's coefficient of correlation, n = 21, and Fig. 2B–C), while after presentation of the movie A, they were not correlated significantly (*p *> 0.05) (Fig. 2A). Increased discomfort after presentation of movies is indicated as the positive values of the differences in scores of the subjective evaluation in Fig. 2.

**Table 3:** Correlation coefficient (Pearson) between CR_ratio _and differences in the scores of questionnaire

	**correlation coefficient**	**level of significance**
**movie A**	0.059	0.799
**movie D**	-0.567**	0.007
**movie R**	-0.590**	0.005

**, *p *< 0.01. In the second column, the levels of significance are shown.

The correlation of the ρ_max_ratio _with the difference in the scores of questionnaire was not significant when the subjects watched any of three movies (*p *> 0.05) (Fig. 2D–F, and Table 4). In addition, the CR_ratio _was not correlated with the ρ_max_ratio _(*p *> 0.05) (Table 5).

**Table 4:** Correlation coefficient (Pearson) between ρ_max_ratio _and differences in the scores of questionnaire. In the second column, the levels of significance are shown

	**correlation coefficient**	**level of significance**
**movie A**	032	0.161
**movie D**	0.16	0.491
**movie R**	-0.42	0.059

**Table 5:** Correlation coefficient (Pearson) between ρ_max_ratio _and CR_ratio_. In the second column, the levels of significance are shown

	**correlation coefficient**	**level of significance**
**movie A**	0.07	0.77
**movie D**	0.11	0.65
**movie R**	0.21	0.37

## Discussion

### Changes in pupillary parameters

The diameter of the pupil is controlled both by sympathetic and parasympathetic activities [13]. Pupil is constricted by increased contraction of the pupillary constrictor muscle, which is innervated by parasympathetic short ciliary and oculomotor nerves, and/or by decreased tension of the pupillary dilator muscles, innervated by sympathetic nerves. The parasympathetic oculomotor neurons are in the dorso-rostral oculomotor nucleus in the midbrain. Sympathetic innervation is originated from the cervical and superior thoracic segments of the spinal cord. Changes in pupillary size may reflect the balance of sympathetic and parasympathetic tones, and are the good measure of the sustained state of the autonomic function. On the other hand, the pupillary light reflex is controlled through the arc through the retinal ganglion cells, the pretectum, and the parasympathetic oculomotor neurons. Changes in parameters of the pupillary light reflex depend probably on the activation levels of the brain stem structures related to this reflex arc.

In point of view of the sustained autonomic function, it is suggested that parasympathetic tone prevailed over sympathetic tone after presentation of any of three movies, because baseline pupillary sizes were decreased (Table 1). The miotic condition may be caused by the relaxed condition of the subject after the end of the task, by the fatigue or by the drowsiness in the dark room, although no subject reported sleepiness in the experiment. On the other hand, CR, which is the change in the amplitude of the light reflex, was different, depending on the movies. The CR increased significantly after presentation of two CG movies, and the change was not significant after presentation of the movie R (Tables 1). Because changes in baseline pupil diameters were not significantly different among three movies, the differences in CR were not dependent on the mean brightness of movies, and other causes should be sought. By comparing properties of three movies (Material and Methods, presentation of motion pictures), changes in the CR might be induced by accumulation of the activities in the brain stem possibly due to the unnatural changes in the disparity and/or brightness, which could facilitate the transmission of the visual signals to the intraocular sphincter muscles.

### Changes in the cardiovascular reflex

Cardiovascular measures, such as spectral analyses of the R-R interval in the cardiac rhythm [14-16], have been typical tools to evaluate the autonomic nervous function. However, in these traditional methods, only slight body movement was allowed. By newly developed index, the ρ_max_, stable measurement of parameters of the cardiovascular reflex is possible when the subjects watch movies with less severe restriction of body movement.

The ρ_max _was increased significantly after presentation of the movie D. In movie D, subjects were met various dinosaurs one after another and were attacked by some of them, which drove cardiac reactions to escape from them. Such reactions could increase the contribution of the baroreflex over biological noises (see Materials and Methods, measurement of blood pressure and ECG), which might increase ρ_max_.

### Correlation among the pupillary, cardiovascular and subjective indices

In order to relate the subjective and objective evaluation of biomedical effects induced by presentation of images, changes in the CR and ρ_max _were related with the differences in the scores of questionnaire. Differences in the subjective evaluation correlated significantly with changes in CR after presentation of movies D and R, but in other combinations the correlation was not significant (*p *> 0.05).

The different relation to the subjective evaluation of the pupillary light reflex and the baroreflex would suggest that different factors contributed to the biomedical influences caused by image presentation. Although further studies with larger number of subjects are necessary, it is suggested that biomedical influences should be evaluated by multiple physical parameters, which are carefully selected.

### Significance of the present study

Pupillary and cardiovascular parameters as well as subjective evaluation were changed after image presentation, and the effects were different depending on the types of images. The results may be utilized to detect subtle changes in physical parameters to assess the effect of medical care [17,18]. Images can also be used as the tool for the treatment, for example, of the patients with panic disorder [19]. Images should be carefully selected, however, and the biomedical effects must be carefully monitored to avoid side effects or aggravation. Rehabilitation of posture and movement of paralyzed patients may be facilitated in virtual environment which would promote their motivation, but some patients may complain of cybersickness due to the virtual motion scenery. Tools to monitor biomedical influences are also needed, which are provided by monitoring parameters such as shown in the present study.

Although many questions remained to be clarified, this study is an important step to accumulate knowledge on biomedical effects evoked by audiovisual stimulation. By accumulation of such knowledge, the efficient tools would be developed to select proper images applicable to the medical care and rehabilitation, and to monitor undesirable effect of images to avoid side effect.
